# Cardiovascular Complications in Systemic Lupus Erythematosus

**DOI:** 10.7759/cureus.26671

**Published:** 2022-07-08

**Authors:** Rahmah Alghareeb, Afshan Hussain, Marvi V Maheshwari, Nabeeha Khalid, Pragnesh D Patel

**Affiliations:** 1 College of Medicine, University of Baghdad, Baghdad, IRQ; 2 Research, Dow Medical College and Dr. Ruth K. M. Pfau Civil Hospital Karachi, Karachi, PAK; 3 Research, Our Lady of Fatima University College of Medicine, Valenzuela, PHL; 4 Cardiology, Omar Hospital and Cardiac Center, Lahore, PAK; 5 Research, St. George's University School of Medicine, St. George's, GRD

**Keywords:** metabolic syndrome, carditis, disrupted immune system, endothelial dysfunction, systemic lupus erythematosus

## Abstract

Systemic lupus erythematosus (SLE) is an auto-immune disease of a relapsing-remitting nature that can cause multiorgan damage depending on several factors, mainly the disease activity. Young age women are the most likely to be affected by the disease and the female-to-male prevalence ratio is approximately 1:10. As the number of SLE patients has been increasing in the last few decades, the annual number of deaths due to the disease and its complications has increased as well, and one of the most important systems to which high mortality is attributed is the cardiovascular system, leading to premature atherosclerosis and other events such as endocarditis and valve disease. In addition to the classical cardiovascular risk factors, studies have found a positive correlation between SLE and other cardio-harmful diseases such as metabolic syndrome and dyslipidemia. Moreover, some of the medications used in the treatment of SLE place a heavy burden on the heart. The article reviews the shared pathophysiology of SLE and cardiovascular disease along with the most common SLE- associated cardiac risks, events, and management.

## Introduction and background

Systemic lupus erythematosus (SLE) is an autoimmune disease that can affect multiple organs in the human body with various clinical manifestations and different severity levels (mild, moderate, and severe) [1]. The term lupus was used for the first time to describe a skin lesion in 916 AD while a clear description of the lupus erythematosus was made by Biett in 1833. At that time, it was reported using the term erythema centrifugum. However, a more obvious picture of SLE and its associated symptoms has been created since the beginning of the 20th century [2]. It is estimated that the disease is highly prevalent in North America (241/100000 people (95% CI: 130, 352)) and least prevalent in Northern Australia (0 cases in a sample of 847 people), moreover, it is found that mortality is higher in SLE patients, in comparison with the general population, with infection and cardiovascular diseases being the most common causes of death [3-4]. Being predominant in young age females, the disease peaks around the age of middle adulthood while appearing in men later in life. Furthermore, being of a black ethnic group increases the chances of having the disease in contrast to Caucasian ethnicity [3]. Although many studies have been dedicated to getting more knowledge about SLE over the years, risk factors are still vague; anyhow, it is believed that the disease depends on multiple factors, rather than one, for its development, especially genetic and environmental ones [5]. As well as that, it is suggested that the initial step in the progression of the disease is a change in the programmed cell death (apoptosis) that leads to the deposition of cell remnants in different organs in the body along with an increase in the activity of B and T lymphocytes. Additionally, neutrophil-specific kind of death triggers the formation of autoantibodies against nuclear antigens leading to immune complex deposition, inflammation, and organ damage [1,6]. Due to its ability to make an impact on multiple systems in the body, SLE patients present with various clinical features, mainly, constitutional symptoms, such as fatigue and fever, mucocutaneous lesions, and musculoskeletal manifestations; for example, arthralgia and arthritis. It is important to mention that about half of SLE patients suffer from blood and neuropsychiatric disorders in addition to the involvement of many other organ systems in the body as the heart, lungs, eyes, kidneys, and gastrointestinal tract; also, SLE is associated with the antiphospholipid syndrome, which increases the risk of fetal loss in pregnant women [1]. The diagnosis of SLE relies on a combination of clinical symptoms, laboratory tests, and tissue biopsy, however, the detection of autoantibodies is of great significance in reaching the right diagnosis such as anti-nuclear antibody (ANA), which is highly sensitive, anti-double-stranded DNA (anti-Ds-DNA) and anti-Smith antibodies, which are highly specific, anti-Sjögren's-syndrome-related antigen A (anti-SSA) and anti-Sjogren's syndrome B (anti-SSB) autoantibodies, as well as a decreased level of C3 and C4 complements. One of the pivotal steps to managing SLE is through lifestyle modification, patient education regarding the disease`s pathogenesis, and the importance of compliance with medications to achieve remission. Additionally, the type of medication modality to be used depends upon the organ system involved with SLE and the severity of tissue damage, thus, a wide range of drugs are used, most commonly, the antimalarial drug hydroxychloroquine, which enables the patients to reach the remission state, moreover, glucocorticoids and immune-suppressive treatments are frequently used to treat SLE [1,7]. The involvement of the heart in SLE includes many conditions, such as pericarditis, which is the most common, valve diseases, coronary artery diseases due to premature atherosclerosis, and heart failure, which carries a high rate of mortality when compared to non-SLE patients [8-9]. This review article aims to highlight the association between SLE and the accompanying heart and vascular diseases by discussing the pathophysiology of SLE and underlying beneficial screening tests and treatment modalities for a variety of heart problems that occur as a consequence of SLE.

## Review

SLE is a disease of loss of self-tolerance with a prevalence of about 1 in 1000 though the scientific progress that has been witnessed in the last few decades has introduced medications that helped improve the quality of life and increase the life span of SLE patients. As death from disease activity has decreased, death from cardiovascular diseases (CVDs) has become more prominent [10].

Pathophysiology

Cardiovascular involvement in SLE is the consequence of a combination of pathogenic mechanisms that work in synchronization, leading to the development of many cardiac events at a younger age compared to the general population [11]. Asanuma et al. published a study in 2003 where the sample population was 134 participants of the same race, age, and sex: 65 patients with SLE (mean age 40.3 years) and 69 controls (mean age, 42.7 years) with no known medical history of coronary artery disease. Electron beam computed tomography was used in the study to check for the availability of coronary artery calcification, and when found, the degree of calcification was measured using the Agatston score. The results of the study showed that coronary-artery calcification was more common in SLE patients (20 of 65 patients) in comparison to controls (6 of 69 subjects) (P=0.002). The mean calcification score was 68.9+/-244.2 in the patients and 8.8+/-41.8 (P<0.001) in the controls, and the study concluded that the prevalence of coronary artery atherosclerosis increases in SLE patients and occurs among them at an earlier age [12].

Endothelial Cells Dysfunction

It's suggested that the origin of atheroma is abnormalities in endothelial cell activation, a process that facilitates the secretion of chemokines, which attract monocytes and enhance their migration through blood vessel walls, and the formation of the oxidized low-density lipoprotein (oxidized-LDL) receptor: lectin-like oxidized low-density lipoprotein receptor 1 (LOX-1). LOX-1 is a pro-inflammatory receptor that increases the CVDs in SLE by triggering the secretion of tissue necrotic factor-alpha (TNF-a), interleukin6 (IL6), and interleukin12 (IL12), which play vital roles in the recruitment of monocytes to adhere to the endothelial cells. Besides that, endothelial adhesion molecules, such as intercellular adhesion molecule 1 (ICAM-1), vascular cell adhesion molecule 1 (VCAM-1), and E-selectins that encourage monocyte adherence to endothelial cells were found to be at higher levels in SLE patients [11,13-15]. Kluz et al. conducted a study in 2009 among a sample population of 67 women (51 SLE cases and 16 healthy controls). The cases were subdivided according to their SLE Disease Activity Index (SLEDAI) score into two subdivisions. The first one included patients with severe SLE activity developing vascular complications while the second included patients with mild to moderate SLE activity with no apparent vascular complications. Then, the serum levels of several adhesion molecules, as well as apoptotic circulating endothelial cells (CECs), were measured using enzyme-linked immunoassay (ELIZA). The study results had shown that the apoptotic CECs were significantly high only in the first subgroup in comparison to healthy controls, serum VCAM-1 was found to be higher in the first subgroup than in the second and its levels in the second were more than that of healthy individuals, however, serum ICAM-1 and E-selectin were increased in both subgroups when compared to healthy participants, thus the study concluded that the serum levels of VCAM-1 is related to the activity of SLE and the development of lupus microvascular events [16]. Once monocytes reach the intima of the blood vessel, they transform into macrophages that engulf the oxidized-LDL, making foam cells (FCs). These FCs are the cornerstone element for plaque formation. At the same time, it is found that monocytes are available in higher numbers than usual in SLE patients [14].

Disrupted Innate Immune System

When talking about innate immunity, it is important to mention that several cardiac problems, such as blood vessel inflammation and stiffness, unstable coronary plaque, and thrombus formation, are the result of the disrupted function of neutrophils and their subclass low-density granulocytes (LDGs), a process that accompanies SLE [17]. Moreover, LDGs trigger the formation of neutrophil extracellular traps (NETs), which are mesh-like fibers with a unique consistency of deoxyribonucleic acid (DNA), histones, and myeloperoxidase (MPO). Together with neutrophils, NETs are found in the lumen and the walls of blood vessels performing an antimicrobial function in normal conditions. However, when their activities become dysregulated due to SLE, they facilitate endothelial damage and subsequent clot creation [18-19]. Dendritic cells (DCs) are antigen-presenting cells that belong to the innate immune system and represent the link between the innate and adaptive immunities. In healthy people, these cells get activated forming plasmacytoid dendritic cells (pDCs), which are responsible for type 1 interferons (IFNs) secretion, while in SLE patients, defective pDCs activation by self-nucleic acids leads to excessive secretion of type 1 IFNs that contribute to cardiovascular problems through multiple mechanisms: disrupting the equilibrium between endothelial damage and vascular repair, decreasing the quality of endothelial progenitor cells (EPCs), increasing the synthesis of many interleukins and cytokines that have pro-inflammatory impacts, participating in foam cells formation while impairing smooth muscle maturation encouraging plaque rupture and enhancing pro-thrombotic events via platelet activation. All these effects of exaggerated IFN levels promote accelerated atherosclerosis in SLE patients [20-22]. Natural killer (NK) cells, the lymphocytes of the innate immune system which link it to the adaptive one, face loss of their cytotoxic effects in SLE due to the downregulated expression of CD3ζ, an important molecule for NK cells activation. The number of NK cells in blood is inversely related to disease activity, and these cells involve in a bi-directional interaction with pDCs resulting in the production of inflammatory cytokines and chemokines, including interferon-alpha (IFN‐α), interferon-gamma (IFN‐γ), tumor necrosis factor-alpha (TNF‐α), IL‐6, IL‐8, chemokine C-C motif ligand 3 (CCL3), and chemokine C-C motif ligands 4 (CCL4). With these pro-inflammatory effects, as well as perforin and granzyme B production, NK cells pave the road for atherosclerosis development in SLE patients [23-25].

Disrupted Adaptive Immune System

Regarding the adaptive immune system, both B and T lymphocytes make a significant contribution to the pathogenesis of SLE alongside autoantibodies [26]. One of the most obvious features that assist in the progression of CVD in SLE is the over-activation of T lymphocytes, including CD4+T cells, especially when differentiated to T-helper 1 (Th1) and T-helper 17 (Th17), which promote vascular injury and clot formation under the control of IFN-1 signals [27]. T-regulatory (Treg) cells, a subgroup of CD4+T cells, are known to be of athero-protective benefits due to their function in diminishing the lymphocyte’s self-reactivity. However, Treg cell dysfunction, which is associated with SLE, results in the loss of their protective cardiovascular merits [22,28]. The hyperactive T cells become less responsive to Treg suppression because of the decrease in IL-10 receptor expression, and therefore, a significant increase in IL-10 facilitates endothelial tissue damage [27]. B lymphocytic cells have two main subgroups: B1 cells, which secrete cardio-protective IgM antibodies that bind the harmful oxidized-LDL and apoptotic cells, and B2 cells, to which the follicular and marginal zone B cells belong, which promote atherogenic activities [29]. However, there is a loss of the cardio-protective features of B1 cells in SLE patients due to the absence of secreted IgM antibodies [30]. Generally speaking, allowing the body’s immune system to attack itself in SLE enhances the production of multiple autoantibodies, most importantly, antiphospholipid (aPL) antibodies that present in about one-third of the cases of SLE while around 10-15% of cases with SLE present with the clinical features of secondary antiphospholipid syndrome (APS) [31-32]. APS is a pro-thrombotic disease that is characterized by periodic arterial or venous thrombosis as well as miscarriage. The disease is associated with abundant serum IgG or IgM anti-cardiolipin (aCL) antibodies and lupus anticoagulant (LAC) antibodies. It is subdivided into primary and secondary APS, whereas the latter correlates with SLE and is linked to the increased CVDs risks in SLE [33].

**Figure 1:**
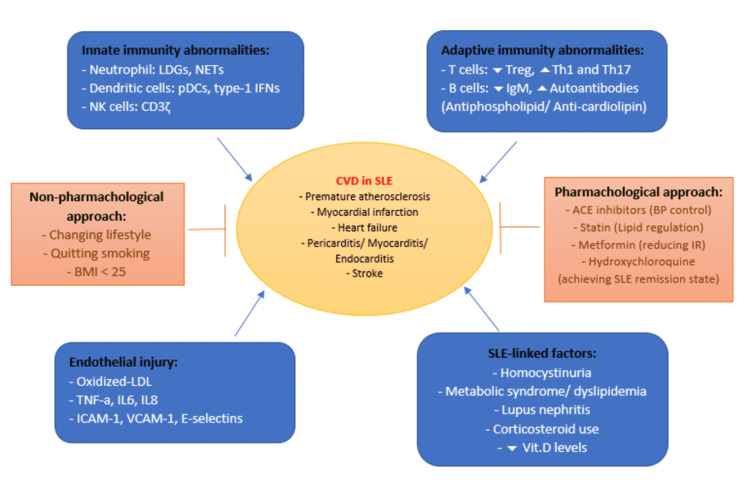
Summary of cardiovascular events, risks, pathophysiologic mechanisms, and treatment in SLE SLE: systemic lupus erythematosus; CVD: cardiovascular disease; LDGs: low-density granulocytes; NETs: neutrophil extracellular traps; pDCs: plasmacytoid dendritic cells; IFNs: interferons; NK cells: natural killer cells; Treg: regulatory T cells; Th1: T helper 1; Th17: T helper 17; BMI: body mass index; ACE inhibitors: angiotensin-converting enzyme inhibitors; BP: blood pressure; IR: insulin resistance; LDL: low-density lipoprotein; TNF-a: tissue necrotic factor-alpha; IL6: interleukin 6; IL8: interleukin 8; ICAM-1: intercellular adhesion molecule 1; VCAM-1: vascular cell adhesion molecule 1; vit.D: vitamin D Image Credit: Rahmah Alghareeb

Figure 1 illustrates the most important pathophysiologic mechanisms for CVDs in SLE.

CVD mortality in SLE

Death due to CVDs in SLE patients is recognized to occur at two peaks; the earlier one is connected to disease activity and its adverse consequences while the late peak is related to atherosclerosis [34].

Clinical manifestations of CVD in SLE

Cardiac Risks and Events

Pericarditis, myocarditis, valve diseases, and conducting system problems are among the most common cardiac outcomes in SLE patients [35]. The pericardium is one of the most commonly involved sites in SLE with a prevalence of about 62% according to some postmortem examination studies [36]. Smiti et al. worked on a retrospective study in 2009 of 97 SLE Tunisian patients who were diagnosed between 1987 and 2005 and had undergone echocardiography evaluating them for pericarditis. The study showed that 38 patients suffered from pericarditis with an average age of 36.4 years while 59 patients with no pericarditis were detected and their average age was 28.8. The conclusion of the study was that pericarditis is the most common cardiac correlated event in SLE and it is associated with pleuritis while anticardiolipin antibodies are strongly positive with the involvement of the heart valves and seem to be less common in pericarditis [37].

In spite of not being that common, lupus myocarditis represents a serious cardiac implication due to its effects on the conducting system and function of the heart [38]. The disease activity and ethnicity play a role in the progression of myocarditis in SLE, which is detected by echocardiography, cardiac MRI, or endomyocardial biopsy in the presence of a high index of suspicion [39-40]. A multicenter study that was published in 2017 by Thomas et al. found that among the 29 SLE patients that were chosen for the study (with a median age at the time of SLE diagnosis of about 30 years), 17 had myocarditis as the first manifestation for SLE and 19 had a less than 45% left ventricular ejection fraction (LVEF), which began to improve after starting the immunosuppressive treatment, leading to a conclusion that myocarditis in SLE is the consequence of lack of the proper medications [41].

Libman-sacks endocarditis (LSE) is a non-infectious lesion that occurs concurrently with SLE and antiphospholipid syndrome targeting heart valves, especially the mitral valve, causing valve stenosis or regurgitation [42]. In addition to aPL autoantibodies, the presence of LSE is associated with NETs, which have been found to have lupus auto-antigens such as histones and (ds)DNA in their composition [43]. Doppler echocardiography was used to evaluate the heart of 342 SLE patients (297 females and 45 males) by Moyssakis et al. in 2007. In those patients, LSE was reported in 38 patients. Twenty-four out of 38 patients were diagnosed with mitral valve regurgitation (MVR), and nine out of the 24 suffered from accompanying mitral stenosis. However, 13 out of the 38 LSE patients had their aortic valve involved while the 38th patient was found to have mild tricuspid regurgitation (TR). Reevaluation included 252 out of 342 patients four years later. It was found that seven out of the 38 patients who were diagnosed with LSE at the beginning had their heart valve condition progress in addition to the death of two patients who were in need of surgery. But eight out of 213, who did not develop any valve lesion at the beginning developed LSE. To sum up the study, one of every 10 SLE patients had LSE with a correlation with lupus duration, disease activity, anticardiolipin antibodies, and antiphospholipid syndrome [44].

The inflammatory nature of SLE is a crucial factor in developing heart failure (HF) [45]. In their retrospective cohort study that was published in 2017, Kim et al. found that SLE puts the heart at a greater danger of the progress of HF in comparison with common people [46]. Another study, a comparative one, which was conducted recently using the information data of Medicaid patients in the United States between 2007 and 2010, found that the number of new cases of HF in SLE patients was about three times higher than the general Medicaid people, and this number was about the same as the incidence of HF in DM [47].

Vascular Risks and Events

Myocardial infarction (MI) is one of the most obvious coronary artery outcomes, which is more likely to occur in SLE patients due to the disease`s pathophysiology. In a retrospective analysis regarding patients with acute myocardial infarction (AMI), it was concluded that patients with AMI and SLE at the same time suffer from higher rates of hospital readmission, post-MI, inpatient death, acute renal injury, and sepsis in comparison to AMI without SLE [48]. According to a published meta-analysis study in 2015, the risk of both types of cerebrovascular accidents, ischemic and hemorrhagic, is increased in SLE patients in comparison to the general population [49]. A systematic review and meta-analysis were established by Yazdany et al. in 2020 based on 26 cohort and cross-sectional studies on MI and stroke. The pooled risk ratio was 2.99 (95% CI 2.34 to 3.82; I2 85%) for MI and 2.18 (95% CI 1.78 to 2.67; I2 75%) for ischemic stroke. The study summarized that SLE patients' likelihood to get MI and stroke is two to three times higher than the general population [50].

An aortic aneurysm (AA) was found to occur in a higher proportion of SLE patients compared to age-matched and sex-matched controls in a study that was published in the second half of the last decade. Although it is not common, it is considered a dangerous complication that must be taken into consideration [51]. Moreover, the development of aortic aneurysm and dissection is associated with old age, male sex, hypertension, and more than three years of SLE duration according to a retrospective cohort study of 15,209 patients with SLE [52].

Hypertension (HT) is an essential predisposing factor for CVDs, and it is highly common in autoimmune diseases that have an impact on the kidneys such as SLE [53]. Sabio et al. worked on a study in 2011 that included 335 women (112 with SLE and 223 healthy controls). The cases and controls were matched for age and each of them was divided into two subgroups depending on their age, younger or older than 40 years old. In the study, a comparison was made between SLE patients with high blood pressure and normal blood pressure levels in terms of inflammatory markers and SLE-associated variables, and it concluded that hypertension was more prevalent in SLE patients when compared to healthy controls (56% vs 29%; p < 0.001) and proportionally higher in SLE women in the younger age group [54].

Other Events and Risk Factors

Homocysteine levels have an impact on blood pressure, particularly, systolic blood pressure (SBP); therefore, they are related to CVDs by causing arteriolar construction, artery stiffness, and kidney dysfunction [55]. Regarding that, a cross-sectional study was done by Sabio et al. in 2014 in which participants were 200 females (99 cases of SLE with no CVDs or diabetes and 101 matched controls). The results found that homocysteine levels (mean ± SD 12.3 ± 4.8 versus 9.3 ± 3.8 μmoles/liter), carotid-femoral pulse wave velocity (PWV), a screening test that measures arterial stiffness, (mean ± SD 7.54 ± 1.1 versus 7.10 ± 1.1 meters/second), SBP (mean ± SD 119 ± 13 versus 115 ± 12 mm Hg), hyperhomocysteinemia occurrence (23% versus 7%) and HT (43% versus 12%) were clearly up in SLE participants (P < 0.050 for all). Additionally, the study found a positive association between homocysteine levels, SBP, and PWV in SLE cases but not in controls, and it was concluded that there is a positive relationship between homocysteine and hypertension in SLE [56].

Metabolic syndrome (MetS), which is strongly associated with insulin resistance, obesity, and hypertension, is a known risk factor for CVDs; however, researchers have discovered an increase in the prevalence of MetS with SLE, as well as insulin resistance. This is attributed to the inflammatory nature of SLE and the use of corticosteroids as one of the important therapeutic modalities [57-58]. Kuo et al. performed a systemic review and meta-analysis in 2020 with a total population of 4460 SLE patients who showed, when compared to controls, to have greater values of homeostasis model assessment for insulin resistance (HOMA-IR), standardized mean difference (SMD) of 0.425; 95% confidence interval (CI) 0.156-0.693; I2=93.8%, and higher levels of adiponectin (SMD=0.547; 95% CI 0.219-0.874; I2=90.1%), leptin (SMD=0.843; 95% CI 0.454-1.231; I2=94.4%), and resistin (SMD=0.856; 95% CI 0.199-1.513; I2=96.6%) with an association between serum resistin and SLE activity [59].

Vitamin D is considered an immune-modulator with an impact on innate and adaptive immune systems; also, its decreased level, especially the anti-inflammatory D3, is linked to many autoimmune diseases including SLE. Moreover, it represents a risk factor for MetS [57,60-61]. Chew et al. published in 2021 a multicenter study of newly-diagnosed SLE patients (less than 15 months) who were followed up since the year 2000. The study found that out of 1847 SLE patients, 1163 (63%) had vitamin D measured and 398 (34.2%) individuals were in the lowest 25-hydroxyvitamin D (25 (OH) D) levels, and MetS was detected in 286 of 860 (33%). Additionally, MetS, HOMA-IR, HT, hypertriglyceridemia, and decreased high-density lipoprotein (HDL) were obviously linked to lower 25(OH) D levels according to the study; therefore, it concluded that vitamin D depletion in SLE patients is correlated with CVDs [62].

As it occurs occasionally with SLE, dyslipidemia is a condition of abnormal metabolism of lipids that is considered a risk factor for CVDs due to premature atherosclerosis [63]. In his study of 78 age-matched subjects, 26 SLE patients with CVDs as cases, 26 SLE patients without CVDs as controls, and 26 population-based controls, Hua et al. found in 2009 that the very-low-density lipoprotein (VLDL) was more abundant in SLE cases when compared to SLE controls and more in SLE patients than in controls while the contrary was regarding high-density lipoprotein (HDL) levels. As it was found to be more widespread in controls than in SLE patients and more prevalent in SLE controls than in SLE cases [64].

End-stage renal disease due to lupus nephritis and systemic corticosteroid usage in the treatment of autoimmune disease as SLE are two essential factors connected to CVDs in SLE [65-66]. According to Ajeganova et al. who performed a study in 2020 focusing on 151 people (77 patients and 74 controls, matched for age and sex with the patients' mean age 47 years and 90% were females and controls' main age 51 years and 92% were females), both groups had mild SLE activity and underwent assessment over seven years regarding carotid intima-media thickness (cIMT) with inclusion factors of dyslipidemia, lower levels of HDL, and carotid plaque in patients and controls. Higher systolic blood pressure, total cholesterol:HDL and LDL:HDL ratios, and triglycerides in patients were associated with cIMT progression and concluded that the risk of cIMT in SLE is escalated in the same manner as the general population but the progression is prompted when with the presence of lupus nephritis or using steroids as a treatment for SLE [67].

Table 1 presents a summary of studies on the most prominent cardiovascular risks and events in SLE.

**Table 1:** Summary of the most prominent cardiac and vascular events in SLE SLE: systemic lupus erythematosus; LSE: Libman-sacks endocarditis; MRV: mitral valve regurgitation; RR: relative risk; CI: confidence interval, MI: myocardial infarction; HF: heart failure; LVEF: Left ventricular ejection fraction; I2: heterogeneity score

Reference	Year	Design	Cases/Controls		Findings
Moyssakis et al. [44].	2007		342 SLE patients underwent Doppler echocardiography evaluation and re-evaluation after 4 years.		LSE was reported in 38 patients. 24 out of 38 were diagnosed with MVR, and 7 out of the 38 got their condition progressed upon reevaluation.
Smiti et al. [37].	2009	Retrospective study	97 SLE patients diagnosed between 1987 and 2005 underwent an echocardiography screening.		Echocardiography showed that 38 out of 97 SLE patients suffered from pericarditis with an average age of 36.4 years.
Sabio et al. [54].	2011		335 women, matched for age (112 with SLE and 223 healthy controls).		HT was found to be more prevalent in SLE patients compared to controls (56% vs 29%; p < 0.001) and proportionally higher in younger SLE women.
Wang et al. [52].	2014	nationwide population-based study	15,209 patients with SLE, women about 90% 0f the patients with a mean age of 38 years.		20 SLE patients were found to have aortic aneurysms and 13 developed aortic dissection with an incidence rate of about 3.34 (95% CI, 1.71-6.91; p < 0.001) in comparison to healthy controls.
Thomas et al. [41].	2016	Multicentre retrospective study	29 SLE patients (with a median age at the time of SLE diagnosis of about 30 years)		17/29 cases have myocarditis as the first presentation for SLE. 20/25 cases have increased troponin levels. 25/28 cases have abnormal echocardiography. 19/29 have low LVEF. 20/29 have pericardial effusion.
Kim et al. [46].	2017	Retrospective cohort study	95,400 SLE patients and 98,900 HF patients with a recent diagnosis.		HF incidence was greater in the SLE patients in comparison with controls (0.97% vs 0.22%, RR: 4.6 (95% CI 4.3 to 4.9)
Yazdany et al. [50].	2020	Meta-analysis	SLE cases and healthy controls from 26 observational studies.		The pooled risk ratio was 2.99 (95% CI 2.34 to 3.82; I2 85%) for MI and 2.18 (95% CI 1.78 to 2.67; I2 75%) for ischemic stroke

Table 2 summarizes the previously mentioned studies regarding the other events and risk factors that accelerate the development of CVDs in SLE patients.

**Table 2:** Summary of studies of other SLE-associated CVD risk factors SLE: systemic lupus erythematosus; CVDs: cardiovascular diseases; VLDL: very-low-density lipoprotein; HDL: high-density lipoprotein; HT: hypertension; SBP: systolic blood pressure; PWV: pulse-wave velocity; HOMA-IR: homeostasis model assessment for insulin resistance; cIMT: carotid intima-media thickness; 25(OH)D: 25-hydroxyvitamin D; MetS: metabolic syndrome; SLEVIC: systemic lupus erythematosus vascular investigation cohort

Reference	Year	Design	Cases/Controls	Population	Findings
Hua et al. [64].	2009		26 SLE patients with CVDs were matched to 26 SLE patients without CVDs and matched to another 26 healthy controls.	Mean age= 52+/-8.2 years.	VLDL levels are positively associated with SLE with being more common in SLE cases than in SLE controls, and more common in SLE patients compared to healthy controls, HDL levels are negatively related to the disease.
Sabio et al. [56].	2014	Cross-sectional study	200 females (99 cases of SLE with no CVDs or diabetes and 101 matched controls).		Hyperhomocysteinemia and HT were found to be higher in SLE cases in comparison to the controls (23% vs 7%) and (43% vs 12%) respectively. A positive correlation between homocysteine and SBP, as well as homocysteine and PWV, were discovered in SLE cases.
Kuo et al. [59].	2020	Systemic review and meta-analysis	4460 SLE patients compared to healthy controls.	From 56 studies, online databases were collected.	SLE patients had higher HOMA-IR. Serum levels of adiponectin, leptin, and resistin were more elevated than the controls.
Ajeganova et al. [67].	2020		151 people (77 patients and 74 controls).	68% and 61% of the original cohort, age-matched, sex-matched population from the SLEVIC cohort.	cIMT development was 0.009 mm/year in SLE cases and 0.011 mm/year in controls, p=0.9, but the progression is prompted by lupus nephritis or steroid use.
Chew et al. [62].	2021	Multicenter study	1847 SLE patients with a diagnosis of fewer than 15 months duration.	Patients were followed up since the year 2000.	Vitamin D deficiency was found in 398 of 1163 SLE patients. MetS was detected in 286 of 860.

Management

The initial step in the management is controlling the modifiable traditional risk factors for CVD, including quitting smoking, having a healthy body mass index (BMI) of less than 25, and performing regular physical activities, which have shown to play an anti-inflammatory role via increasing Treg with IL-6 secretion from muscles that lead to the production of IL-10 and inhibition of IL-1β, as well as keeping blood pressure <130/85 mmHg using angiotensin-converting-enzyme (ACE) inhibitors, which is the first-line treatment modality for hypertensive SLE patients, especially those with lupus-related kidney problems. Angiotensin receptor blockers (ARBs) can be used as an alternate for those who suffer from ACE inhibitors' side effects. However, beta-blockers have the ability to precipitate Raynaud due to the increase in the activity of the alpha 2-adrenergic receptors of the sympathetic nervous system leading to vasoconstriction of the digital arteries [11,14,68-69]. Statins are pivotal in keeping the lipid profile within the normal range, especially LDL, and that was found to be useful not only in decreasing the cardiovascular risks in SLE but also in reducing deaths and end-stage renal disease (ESRD) [12,70]. By down-regulating some of the important inflammatory pathways in SLE pathogenesis and improving insulin resistance, metformin has been shown to decrease the flare-up of mild and moderate severity SLE as well as the associated CVDs [12,71]. The main goal of the SLE treatment regime is to keep the patient in a remission state by reducing disease activity. Hydroxychloroquine is an immune-modulator that represents the standard treatment for all SLE patients, as it has the ability to reduce the flare-up of the disease, other organ involvement, glucocorticoid dose and its morbid side effects, and the thrombotic events of anti-phospholipid syndrome. In addition, it was found that the immune-modulatory effect of antimalarial drugs has atheroprotective effects and improves lipid profile and blood glucose levels [72-74]. According to a systematic review that was done by Ruiz-Irasrorza et al. based on randomized controlled trials and observational studies of anti-malarial drugs, pieces of evidence have shown that hydroxychloroquine is safe, even during pregnancy [75]. Figure 1 illustrates the treatment modality for CVD in SLE patients.

Limitations

The study has not mentioned the diagnostic tools and screening methods of CVDs, focusing particularly on the pathophysiological and clinical manifestations of SLE-associated CVDs.

## Conclusions

SLE is a disease well-known for its cutaneous manifestations, such as malar rash, photosensitivity, and discoid skin lesions; however, it is a multisystemic disease that has the ability to impact every organ system in the body, most importantly, the heart and vascular system, which causes remarkable mortalities among SLE patients, both in the younger and older age groups. Additionally, kidney disease and infection are the other remarkable causes of death in SLE patients. The clinical significance of this review article is to show the intercalation between SLE and cardiovascular diseases, such as premature atherosclerosis and the consequent acute coronary syndrome, heart failure and stroke, and carditis, involving various layers of the heart, with heart valve involvement, which are fatal complications that decrease the life span and quality of life, leading to recurrent hospitalizations, even when the patient is in remission. This article is dedicated to helping understand the connection between SLE and CVDs by focusing on the shared pathological mechanisms of endothelial cell injury, inflammation, and immune system dysregulation, both innate and adaptive, as well as the importance of lifestyle modification as the primary prevention along with immune-modulators as secondary prevention of CVDs that accompany SLE. With the help of the previously mentioned studies, the article has discussed the most common cardiac, vascular, and other SLE-associated events that accelerate heart diseases in SLE such as hyperhomocysteinemia, metabolic syndrome, and lupus nephritis, as well as the medications used in the treatment of the CVDs in SLE while paying attention to the role of immune-suppressants adverse effects in the development of CVDs. In summary, SLE-associated CVD is a serious topic that needs more detailed research, serious efforts, and studies to establish a wider understanding of the various mechanisms and conditions, the prevalence of SLE-related CVDs, triggering factors, clinical presentations, and management approaches with treatment alternatives to provide longevity and better quality of life for SLE patients.
